# Scientific Association at Nashville

**Published:** 1877-07-20

**Authors:** 


SCIENTIFIC ASSOCIATION AT NASHVILLE.—J. B. Lindsley, Secretary, kindly sends us invitation to the American Association for the Advancement of Science, to be held in Nashville, Tenn., August 29th to September 5th.

We regard this organization of the first importance. It should attract the attention and elicit the co-operation of enlightened and progressive minds, particularly in the South, where exists so many latent and undeveloped resources, not only in the agricultural, but in geological, mining, manufacturing, educational, and other departments.

